# Fibroblast Growth Factors and Their Receptors in Major Depressive Disorder: A Scoping Review of Translational Research

**DOI:** 10.3390/ijms27198464

**Published:** 2026-09-23

**Authors:** Gracja Ryznar, Piotr Gałecki, Katarzyna Bliźniewska-Kowalska

**Affiliations:** 1 Department of Adult Psychiatry, Medical University of Lodz, 91-229 Lodz, Poland; piotr.galecki@umed.lodz.pl (P.G.); katarzyna.blizniewska-kowalska@umed.lodz.pl (K.B.-K.); 2 International Doctoral School, Medical University of Lodz, 90-647 Lodz, Poland

**Keywords:** major depressive disorder (MDD), fibroblast growth factors, FGF-2, FGF-9, FGFR1, neuroplasticity, antidepressants, biomarkers

## Abstract

Major depressive disorder (MDD) is a complex psychiatric condition whose pathophysiology extends beyond the classic monoamine hypothesis, involving impaired neuroplasticity, neuroinflammation, HPA axis dysregulation, and altered neurotrophic signaling. The fibroblast growth factor (FGF) system, comprising ligands and their receptors (FGFRs), has emerged as a key candidate regulator of these processes. This scoping review qualitatively synthesizes current clinical, postmortem, and preclinical evidence on the role of the FGF system in MDD and antidepressant response. A structured literature search according to PRISMA-ScR was conducted in PubMed/MEDLINE and Scopus without date restrictions. Eligible studies included human (clinical, postmortem), preclinical, and translational data investigating FGF/FGFR expression, signaling, manipulation, or peripheral biomarker associations in the context of depression or antidepressant action. Of 287 unique records screened, 68 met the inclusion criteria; together with 11 studies identified through backward citation searching, 79 studies were included in the qualitative synthesis. This synthesis outlines a bidirectional role for the fibroblast growth factor (FGF) system in major depressive disorder (MDD). Rather than acting as a uniform pathway, different FGF ligands exert opposing regulatory effects on mood, neuroplasticity, and stress responses. FGF-2 exerts protective and neurotrophic effects, while factors like FGF-9 act as endogenous prodepressants. Although circulating biomarkers show heterogeneity, central FGF/FGFR signaling represents a promising target for novel therapeutic strategies and personalized treatment approaches in MDD.

## 1. Introduction

Major depressive disorder (MDD) is a complex psychiatric condition characterized by profound mood and cognitive impairments. Beyond the classic monoamine hypothesis, contemporary research highlights a multifaceted neurobiological etiology.

Central to this understanding is the neuroplasticity hypothesis. MDD is associated with significant structural alterations and synaptic remodeling, particularly a reduced hippocampal volume and morphological shifts within the medial prefrontal cortex [1]. Furthermore, adult hippocampal neurogenesis (AHN) has been implicated in the pathophysiology of depression and in antidepressant action: postmortem studies have shown fewer dentate granule neurons in untreated MDD and increased numbers of neural progenitor cells in antidepressant-treated patients [2,3].

Parallel to structural changes, neuroinflammation plays a key role in the pathogenesis of MDD. Elevated proinflammatory cytokines—including interleukin-1β (IL-1β), tumor necrosis factor-α (TNF-α), and interleukin-6 (IL-6)—disrupt neuronal function and directly contribute to depressive symptomatology [4,5,6].

Furthermore, chronic dysregulation of the hypothalamic–pituitary–adrenal (HPA) axis leads to hypercortisolism, one of the most robust neurobiological hallmarks of MDD. This hyperactivity is largely driven by glucocorticoid resistance (impaired receptor-mediated negative feedback) and further exacerbates structural brain alterations [7,8].

Closely linked to stress signaling is the neurotrophic hypothesis, which posits that stress lowers brain-derived neurotrophic factor (BDNF) levels, causing neuronal atrophy in limbic regions. Antidepressants appear to reverse these deficits by upregulating neurotrophic signaling. Alongside BDNF, other growth factors such as VEGF, GDNF, and particularly the FGF system have emerged as critical neurobiological markers and potential therapeutic targets [9,10]. Whereas BDNF and GDNF have been thoroughly investigated in the context of depressive disorders, relatively few studies have addressed the clinical and functional significance of the FGF family.

To address this gap, the aim of this scoping review is to systematically map and evaluate translational evidence regarding the role of the FGF–FGFR signaling pathways in major depressive disorder, with a primary focus on specific FGF ligands and receptors that hold the strongest empirical support.

## 2. Materials and Methods

In the present review, we mapped and organized the available evidence on the role of the fibroblast growth factor (FGF) system—comprising FGF ligands and their receptors (FGFRs)—in depression, spanning direct clinical and postmortem findings as well as preclinical and translational data. To improve transparency, the literature search and study selection were carried out in a structured manner, as described below.

The review was guided by the following question: Which direct, preclinical, and translational findings describe the involvement of the FGF system in the pathophysiology of depression and in the response to antidepressant treatment? Because relatively few studies address FGF signaling specifically in clinically diagnosed depression, the eligible scope was deliberately kept broad, encompassing human clinical and postmortem studies, animal models of depression and chronic stress, investigations of depression-like behavior and antidepressant action, and reports of FGF/FGFR expression, manipulation, or peripheral biomarker associations.

### 2.1. Search Strategy

Two bibliographic databases were searched: PubMed/MEDLINE and Scopus. The PubMed search combined controlled-vocabulary (MeSH) terms with keywords describing the FGF system and depression:

(“Fibroblast Growth Factors”[MeSH Terms] OR “fibroblast growth factor”[Title/Abstract] OR FGF[Title/Abstract] OR FGFR[Title/Abstract]) AND (“Depressive Disorder”[MeSH Terms] OR “Depressive Disorder, Major”[MeSH Terms] OR depression[Title/Abstract] OR “major depressive disorder”[Title/Abstract] OR MDD[Title/Abstract]).

The Scopus search used an equivalent string restricted to titles and abstracts:

TITLE-ABS(“fibroblast growth factor*” OR FGF OR FGFR) AND TITLE-ABS(depression OR “depressive disorder*” OR “major depressive disorder” OR MDD) AND LANGUAGE(english), limited to articles, reviews, and book chapters.

No date limits were imposed, reflecting the comparatively small volume of relevant literature, and both searches were restricted to English-language publications. PubMed was searched on 21 June 2026, and Scopus on 29 August 2026. The reference lists of the included articles and of relevant reviews were screened manually for additional eligible studies (backward citation searching).

### 2.2. Eligibility Criteria

Records were eligible for inclusion if they:

- Examined one or more FGF ligands or receptors (FGFRs) in relation to depression or depression-like phenotypes;
- Reported original human (clinical or postmortem), preclinical (animal or cellular), or translational data;
- Reported FGF/FGFR expression, pharmacological or genetic manipulation, or peripheral biomarker associations relevant to depression or antidepressant action.

Records were excluded if they:

- Were not concerned with the FGF system in the context of depression or depression-like phenotypes;
- Were editorials or conference abstracts without primary data;
- Addressed the FGF system only as general background (e.g., developmental biology, or reviews centered on other disorders); such sources were retained for the Introduction rather than the evidence base.
- Were narrative reviews without original data; such articles were used for backward citation searching and, where relevant, as background sources, but were not included in the evidence synthesis. Meta-analyses providing pooled estimates were retained.
- Had no retrievable full text or a full text available only in a language other than English.
- Did not report an extractable result for an FGF ligand or receptor in relation to depression or a depression-like phenotype.

### 2.3. Study Selection

No review protocol was registered. The review was designed as a scoping review to map a small and heterogeneous body of literature, and the search strategy and eligibility criteria were specified before screening began. Records from both databases were pooled and de-duplicated. Titles and abstracts were screened against the eligibility criteria independently by two reviewers (G.R. and K.B.-K.). Disagreements were resolved by discussion, and unresolved cases were referred to the senior author (P.G.). Potentially eligible records were then assessed in full text. Data from included studies were charted in a summary table (Table S1) covering study design, population or model, sample size, tissue or compartment, FGF ligand or receptor examined, measurement method, and main findings.

The database searches returned 513 records (268 from PubMed and 245 from Scopus). After removal of 226 duplicates, 287 records were screened by title and abstract, and 198 were excluded as unrelated to the FGF system in depression. Eighty-nine reports were sought for full-text assessment; one could not be retrieved. Of the 88 reports assessed in full text, 20 were excluded: 13 were narrative reviews without original data, one did not concern the FGF system in depression, one had a full text available only in Russian, two did not report an extractable result for an FGF ligand or receptor in relation to depression or a depression-like phenotype, two measured FGF-2 only as one of a broader panel of neuroplasticity-related transcripts in naive animals without a depression-related comparison, and one was an overlapping report of an included study. Backward citation searching of the reference lists of included reports and relevant reviews identified 11 further eligible reports. In total, 79 studies were included in the synthesis (68 from the database searches and 11 from reference lists).

### 2.4. Data Synthesis

Because the available evidence is limited and the included studies differ considerably in design, populations, models, and outcomes, the findings were synthesized qualitatively. The characteristics and main results of the included studies were charted in a summary table to map the nature and distribution of the available evidence.

The selection process is presented in Figure 1, prepared as a PRISMA ScR-style flow diagram.

Table S1 (Supplementary Materials) summarizes included studies.

## 3. Results and Discussion

Overview of the Fibroblast Growth Factor Family and Its Functional Classification (Figure 2).

Fibroblast growth factors (FGFs) constitute a large family of growth factors composed of 22 ligands and 5 receptors [10,11,12]. Based on their mechanisms of action, FGFs are classified into three functional groups: canonical, endocrine, and intracellular FGFs [11,13]. In addition, the FGF family can be subdivided into seven subfamilies. The majority of FGFs, including FGF1–10, FGF16–18, FGF20, and FGF22, are classified as secreted canonical FGFs. These proteins are further organized into five paracrine subfamilies: the FGF1 subfamily (FGF1 and FGF2), the FGF4 subfamily (FGF4, FGF5, and FGF6), the FGF7 subfamily (FGF3, FGF7, FGF10, and FGF22), the FGF8 subfamily (FGF8, FGF17, and FGF18), and the FGF9 subfamily (FGF9, FGF16, and FGF20) [11,14]. Canonical FGFs predominantly function through autocrine or paracrine signaling by binding to and activating high-affinity tyrosine kinase receptors encoded by four genes: *FGFR1*, *FGFR2*, *FGFR3*, and *FGFR4* [11,15]. Another distinct group comprises the endocrine FGFs, also referred to as the FGF15/19 subfamily, which includes FGF19, FGF21, and FGF23 [11,16]. Owing to their relatively low affinity for cognate FGF receptors, endocrine FGFs are able to enter the circulation and exert hormone-like effects on distant target tissues. These factors play a pivotal role in the regulation of systemic metabolic homeostasis [11,17]. The remaining four family members, FGF11–FGF14, form the intracellular FGF11 subfamily, also commonly referred to as the FGF homologous factor (FHF) subfamily [10]. Unlike canonical and endocrine FGFs, these proteins do not participate in classical extracellular receptor signaling. Instead, they function in an intracrine manner within the cell, where they modulate voltage-gated sodium channels and interact with other intracellular molecular partners [11,15].

Fibroblast growth factor receptors (FGFRs) are receptor tyrosine kinases that mediate the biological effects of FGF ligands and comprise four main members: FGFR1, FGFR2, FGFR3, and FGFR4. Although these receptors share substantial sequence homology, they differ in ligand-binding affinity, which contributes to the tissue- and ligand-specific effects of FGF signaling [18]. Structurally, FGFRs consist of three extracellular immunoglobulin-like domains (D1–D3), a single transmembrane region, and an intracellular tyrosine kinase domain [19,20]. The D1 domain and the acidic “acid box” region located between D1 and D2 participate in receptor autoinhibition, whereas D2 and D3 are mainly responsible for ligand binding and receptor specificity [21]. Alternative splicing within the D3 domain of FGFR1, FGFR2, and FGFR3 generates b and c isoforms, producing seven principal FGFR isoforms: FGFR1b, FGFR1c, FGFR2b, FGFR2c, FGFR3b, FGFR3c, and FGFR4 [22,23]. Activation of FGFR signaling requires formation of a complex between FGF ligands, receptors, and cofactors such as heparan sulfate for canonical FGFs or α-/β-Klotho proteins for endocrine FGFs [16,24,25]. Ligand binding induces conformational changes, receptor dimerization, and phosphorylation of the intracellular tyrosine kinase domain, thereby initiating downstream signaling cascades [20].

Canonical FGF signaling is initiated by the binding of an FGF ligand to its cognate FGFR, which leads to receptor dimerization, phosphorylation of the tyrosine kinase domain, and activation of major intracellular pathways, including MAPK/ERK, PI3K/AKT, PLCγ/PKC, and STAT signaling. These pathways regulate processes such as cell survival, proliferation, differentiation, migration, and inflammatory responses. In addition, FGFs may act through non-canonical signaling mechanisms that are independent of FGFR kinase activity or, in some cases, even independent of ligand binding. In this context, signaling is mediated, among others, by syndecans, integrins, neural cell adhesion molecule (NCAM), and N-cadherin, thereby influencing adhesion, migration, angiogenesis, neuronal survival, and neurite outgrowth. Together, canonical and non-canonical signaling expand the functional diversity of the FGF system and enable context-dependent cellular responses [11].

FGF signaling is tightly regulated by cell-surface cofactors and inhibitory feedback mechanisms that determine its intensity, specificity, and duration. In paracrine FGF signaling, heparan sulfate proteoglycans (HSPGs) act as essential cofactors for pathway activation, whereas endocrine FGFs depend on α-/β-Klotho proteins for effective ligand–receptor interaction and FGFR activation. FGFRL1 may further modulate this process by functioning as a decoy receptor or regulator of receptor turnover and signaling. Signal termination and fine-tuning are mediated by negative feedback mechanisms, including ERK1/2-mediated inhibition of FGFR1, intracellular regulators such as SPRY, SEF, DUSP6, GRB14, and SHP2, and CBL-dependent ubiquitination and degradation of FGFR and FRS2α. Together, these mechanisms maintain FGF signaling as a transient, controlled, and context-dependent process [11].

**Figure 2:**
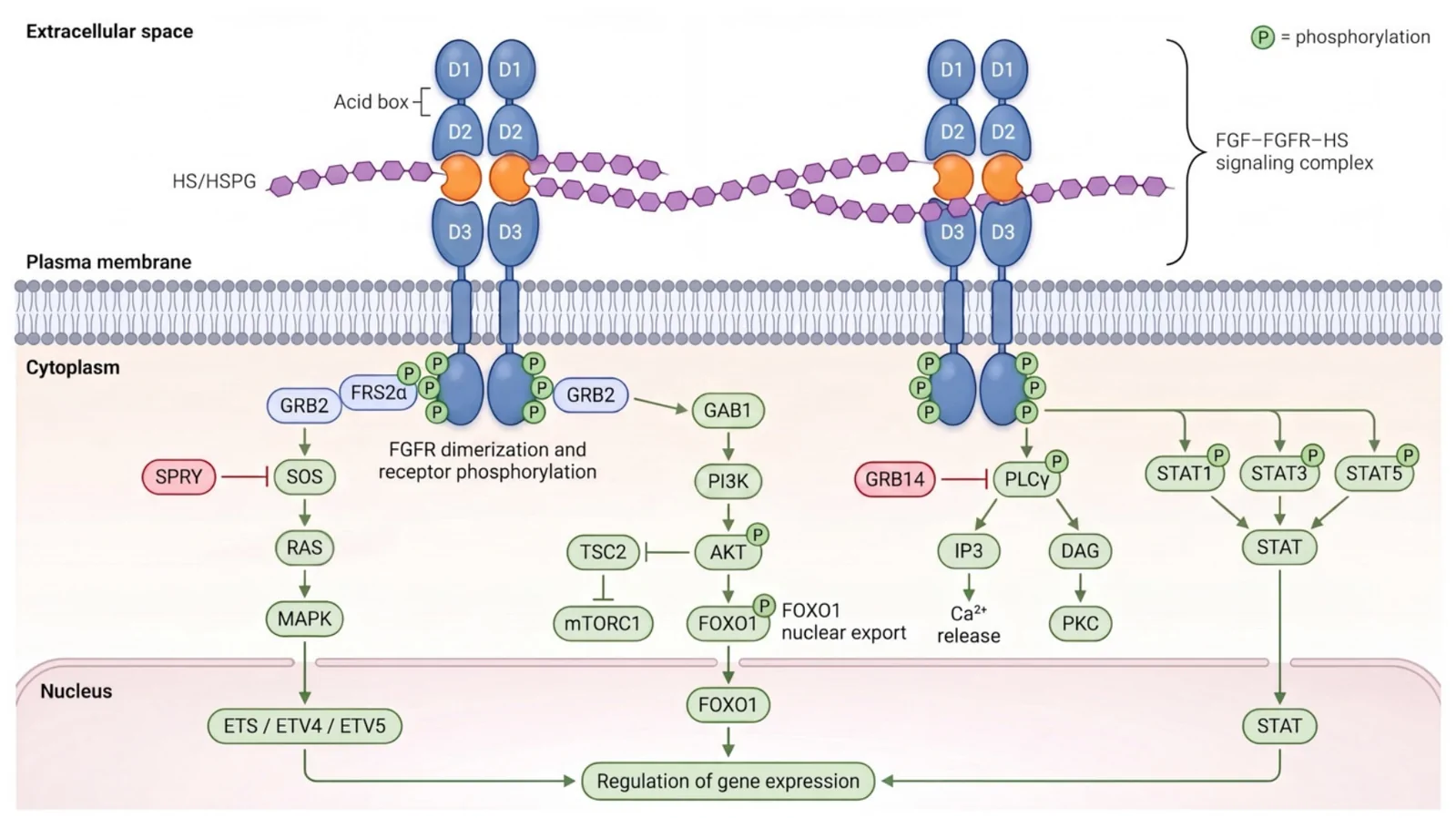
**Canonical FGF–FGFR signal transduction.** A canonical FGF ligand, with heparan sulfate/heparan sulfate proteoglycan (HS/HSPG) acting as a cofactor, binds the extracellular immunoglobulin-like domains (D1–D3) of two FGF receptors to form an FGF–FGFR–HS signaling complex. Complex formation drives receptor dimerization and trans-autophosphorylation of the intracellular tyrosine kinase domains (P). The activated receptor engages four principal intracellular cascades. In the RAS–MAPK pathway, the phosphorylated docking protein FRS2α recruits GRB2 and the exchange factor SOS to activate RAS and the downstream MAPK module, with SPRY acting as a negative regulator; MAPK signaling induces ETS-family transcription factors (ETV4, ETV5). In the PI3K–AKT pathway, GRB2 recruits GAB1 to activate PI3K and AKT; AKT promotes mTORC1 activity by inhibiting TSC2 and phosphorylates FOXO1, driving its nuclear export. In the PLCγ pathway, the receptor activates PLCγ, which generates IP_3_ and DAG, leading to Ca^2+^ release and PKC activation; GRB14 inhibits PLCγ. In the STAT pathway, the receptor activates STAT1/3/5, which translocate to the nucleus. These cascades converge in the nucleus to regulate gene expression. Prepared by the authors on the basis of the mechanisms described in Ornitz and Itoh (2015) [15]. The FGF–FGFR signaling pathway schematic (Figure 2) was first drafted manually by the authors, who designed the scientific content, all pathway components, and the overall layout on the basis of Ornitz and Itoh (2015) [15]. This manual draft was then rendered and refined into the final publication-quality figure using AI-based scientific illustration tools (FigureLabs and SciDraw AI), which were used at different stages of figure preparation. No scientific content was generated autonomously by the tools. All authors reviewed and verified the accuracy of every element of the figure and take full responsibility for its content.

### 3.1. Fibroblast Growth Factor 2 (FGF-2)

FGF-2 is an important neurotrophic growth factor within the central nervous system, where it participates in processes essential for neuronal plasticity. In particular, FGF-2 has been linked to hippocampal neurogenesis, synaptic development, long-term potentiation, learning and memory, as well as neural responses to injury [26,27,28]. The hippocampus appears to be especially relevant in this context, as it shows high expression of FGF-2 and its receptors, supporting the potential involvement of this pathway in depression-related disturbances of hippocampal structure and function [29].

A key question across the analyzed studies was whether peripheral and central FGF-2 levels differ between individuals with depression and non-depressed controls. The results were clearly inconsistent. The data on peripheral FGF-2 in particular indicate substantial discrepancies. A preliminary meta-analysis showed significantly higher levels of peripheral FGF-2 protein in patients with MDD compared with healthy controls (*p* = 0.005). This preliminary analysis pooled four case–control studies with 99 patients and 80 controls and detected no between-study heterogeneity. In the same analysis, postmortem *FGF-2* mRNA did not differ between groups, whereas central *FGFR1* mRNA was higher in MDD [30]. However, a meta-analysis published in 2020 did not find a significant difference between these two groups [31]. A subsequent meta-analysis comprising eight studies with 310 patients and 268 controls likewise found no significant difference in peripheral FGF-2 concentrations between patients with depression and healthy controls (Hedges’ g = −0.288; 95% CI −0.828 to 0.253) [32]. Between-study heterogeneity was substantial and was not explained by sample source, age, sex, symptom severity, or year of publication. The pooled samples were clinically heterogeneous, encompassing treatment-resistant, late-life, and comorbid presentations, and information on medication status, illness duration, and body mass index was largely unavailable. The divergence between the initial meta-analysis and the two subsequent analyses is therefore more plausibly attributable to the limited size and clinical heterogeneity of the evidence base than to a consistent alteration in either direction [32].

Moreover, smaller primary studies included in this review reported decreased serum FGF-2 levels in patients with MDD, further highlighting the inconsistency of peripheral FGF-2 findings [33,34]. Conversely, Kahl et al. reported elevated serum FGF2 concentrations in individuals with depression who also had borderline personality disorder [35]. Lu et al. likewise observed that patients with major depressive disorder and a history of childhood trauma showed significantly higher peripheral blood FGF2 levels compared with healthy controls [36].

In the study conducted by Becerril-Villanueva et al., adolescents with major depressive disorder demonstrated significantly elevated serum levels of basic fibroblast growth factor (FGF-2) compared to healthy controls, with fluoxetine treatment producing a transient decrease at week 4 that returned to baseline levels by week 8 [37].

Lebowitz et al. extended their earlier pediatric findings [34] to 259 mothers and their children, most of whom had been referred for anxiety and/or depressive symptoms [38]. Maternal and child serum FGF-2 concentrations were positively correlated, and lower child FGF-2 was associated with higher self-reported anxiety and depressive symptoms. In a structural model adjusted for maternal symptoms, maternal FGF-2 was not directly associated with child symptoms but was linked to them indirectly through the child’s own FGF-2 levels. Child FGF-2 did not differ between referred and non-referred children after adjustment for demographic covariates. As the sample partly overlapped with the original cohort and the design was cross-sectional, these findings pertain to dimensional symptoms rather than diagnosed MDD and do not permit causal inference [38].

Three studies found no significant differences in peripheral FGF-2 levels between depressed and control groups. The most comprehensive evidence comes from a meta-analysis by Shi et al. (2020), which pooled data from 230 MDD patients and 141 healthy controls and found no significant difference in peripheral FGF-2 levels (pooled SMD = 0.617; 95% CI = −0.255 to 1.490; *p* = 0.166) [31]. Additionally, two population-specific studies found null results: Wu et al. (2019) reported no significant differences in plasma FGF-2 levels between 89 elderly MDD patients and 51 healthy controls, while Takebayashi et al. (2010) found no significant difference in plasma FGF-2 levels between MDD patients in remission and matched control subjects [39,40]. These findings suggest that age and disease state may moderate the relationship between peripheral FGF-2 and depression, with elderly populations and remitted patients potentially not exhibiting the same alterations observed in other demographic groups.

The variability of peripheral findings may partly reflect the acute regulation of FGF-2 by stress. Bryant et al. measured salivary FGF-2 and cortisol in 87 healthy adults exposed to the Trier Social Stress Test and in a separate no-stress group [41]. Salivary FGF-2 increased transiently after the stressor and remained stable without it. The temporal patterns of FGF-2 and cortisol change were associated, but the magnitude of their responses was unrelated, suggesting partly independent components of the stress response. Lower FGF-2 reactivity, but not absolute concentration, was associated with greater repetitive negative thinking and fear of negative evaluation, and predicted subsequent rumination. Although the participants had no psychiatric history, these findings indicate that single-time-point peripheral FGF-2 measurements may be sensitive to the sampling context [41].

In contrast to the mixed findings reported for peripheral FGF-2, postmortem studies appear to provide more consistent evidence of reduced FGF-2-related signaling within the brain of individuals with major depressive disorder. Gaughran et al. reported a significant decrease in *FGF-2* mRNA-positive cell density in the CA4 region of the hippocampus, while Evans et al. identified dysregulation of several FGF-related transcripts in frontal cortical areas, including reduced expression of FGF-2 [42,43]. Similarly, Aurbach et al. observed lower FGF-2 expression across multiple brain regions, such as the frontal cortex, hippocampus, and locus coeruleus, in depressed subjects compared with controls [44]. Importantly, Evans et al. suggested that these transcript alterations were unlikely to be caused by antidepressant treatment, as selective serotonin reuptake inhibitor (SSRI) exposure appeared to attenuate rather than induce the observed dysregulation. The compensatory increase in *FGFR1* mRNA reported by Gaughran et al. may further indicate a biological response to reduced FGF-2 ligand availability [42,43].

Animal models of depression-like states largely support reduced FGF-2 expression in the brain, although the effect may be region-dependent. Central lipopolysaccharide challenges reduced hippocampal FGF-2 protein in rats [45], and peripheral lipopolysaccharide produced a similar reduction in the mouse hippocampus [46]. Chronic unpredictable mild stress decreased FGF-2 in both the hippocampus and frontal cortex of rats [47]. Prolonged, but not brief, chronic restraint stress reduced hippocampal FGF-2 in mice, in parallel with a reduction in neurotrophic factor-alpha1 (carboxypeptidase E), which was shown to upregulate FGF-2 expression in hippocampal neurons [48]. In a rat model of post-stroke depression, FGF-2 protein and mRNA were reduced in the frontal cortex compared with stroke alone, whereas the hippocampal difference did not reach significance [49]. Zhai et al. identified microRNA-497 as a potential upstream regulator of *FGF2*: hippocampal miR-497 was increased in stressed rats, its knockdown attenuated depressive-like behavior, and *FGF2* was validated as a direct miR-497 target in cultured microglia [50]. FGF-2 itself was not measured in vivo in that study.

Moving from baseline alterations in FGF-2 toward the effects of antidepressant treatment, preclinical evidence suggests that chronic antidepressant administration may enhance central FGF-2 expression. In animal studies, this effect has been observed following treatment with several antidepressant drugs and across different brain regions, although its magnitude appears to depend on treatment duration and the specific area examined. Bachis et al. showed that chronic, but not acute, treatment with desipramine or fluoxetine increased FGF-2 immunoreactivity in layer V cortical neurons as well as in hippocampal astrocytes and neurons [51]. Maragnoli et al. reported that combined treatment with fluoxetine and olanzapine markedly upregulated FGF-2 mRNA in the prefrontal cortex, with chronic co-administration extending this effect to other regions, including the hippocampus and striatum [52]. Further evidence for region-specific regulation was provided by Carboni et al., who found that chronic trazodone increased FGF-2 expression in the prefrontal cortex and amygdala, whereas chronic citalopram increased FGF-2 expression mainly in the hypothalamus [53].

Non-pharmacological and plant-derived interventions have shown similar effects. Acute vagus nerve stimulation in rats increased *FGF-2* mRNA in the hippocampus and cerebral cortex, alongside an increase in prefrontal norepinephrine comparable to that produced by venlafaxine [54]. In rats exposed to chronic unpredictable mild stress, three weeks of high-frequency repetitive transcranial magnetic stimulation improved depression-like behavior and increased FGF-2 and FGFR1 expression in the hippocampus and frontal cortex [47]. In mice, pretreatment with the flavonoid glycoside liquiritin attenuated lipopolysaccharide-induced depressive-like behavior and reversed the associated reduction in hippocampal FGF-2, as did fluoxetine in the same model [46]. In the latter two studies, however, the involvement of FGF-2 signaling was inferred from parallel behavioral and molecular changes rather than tested by blockade of the pathway.

Taken together, these findings suggest that antidepressant treatment may modulate central FGF-2 signaling in a brain-region-specific manner. In line with this interpretation, Evans et al. observed in human postmortem tissue that SSRI treatment appeared to attenuate, rather than induce, the dysregulation of FGF-related transcripts found in untreated individuals with MDD [43]. Conversely, in *Fgf2*-knockout mice, fluoxetine did not reverse chronic-stress-induced depressive-like behavior, suggesting that FGF-2 is required for its behavioral effects [55].

Beyond changes induced by conventional antidepressant treatment, several animal studies have directly investigated whether exogenous FGF-2 administration itself can produce antidepressant-like effects. Overall, these studies suggest that direct administration of FGF-2 may produce antidepressant-like effects across different experimental paradigms. Turner et al. showed that intracerebroventricular administration of FGF-2 reduced immobility in the forced swim test, while repeated FGF-2 treatment decreased depression-like behavior in additional behavioral assays [56]. Jarosik et al. reported that FGF-2 produced antidepressant-like effects in the tail suspension test and reversed depressive-like behaviors induced by olfactory bulbectomy. These behavioral improvements were accompanied by restoration of hippocampal neurogenesis and reduced neuronal loss in brain regions such as the piriform cortex and amygdala [57]. Elsayed et al. further demonstrated that chronic intracerebroventricular FGF-2 infusion prevented anhedonia-like behavior induced by chronic unpredictable stress, whereas subchronic infusion into the prefrontal cortex, but not the dorsal striatum, produced antidepressant- and anxiolytic-like effects [58]. In addition, Wang et al. showed that peripheral administration of both high- and low-molecular-weight FGF-2 attenuated depression-like behaviors in mice exposed to chronic unpredictable mild stress, with high-molecular-weight FGF-2 showing a stronger effect on hippocampal BDNF activity [59]. Similarly, in neurotrophic factor-alpha1-knockout mice, which show reduced hippocampal FGF-2 and neurogenesis together with depressive-like behavior, FGF-2 administration reversed the behavioral phenotype [48]. Exogenous FGF-2 also appears to produce antidepressant-like effects in an inflammatory model. In rats challenged with central lipopolysaccharide, intracerebroventricular FGF-2 reversed depressive-like behavior and partially restored hippocampal neurogenesis, without affecting behavior in non-inflamed animals [45].

Mechanistically, these effects have been linked to activation of ERK1/2 and AKT signaling, increased Bcl-2 expression, inhibition of caspase-3 activation, and restoration of stress-related impairments in BDNF and FGFR1 phosphorylation [45,59,60].

FGF-2 appears to be a potential biomarker and predictor of treatment response in depression, although findings remain inconsistent. Xu et al. (2019) reported that FGF-2, together with BDNF, TNF-alpha, and 5-HT, predicted escitalopram efficacy with high performance (AUC = 0.929; accuracy = 88.7%) [61]. In a separate cohort of 59 patients with MDD and 61 healthy controls, serum basic FGF was among the cytokines that discriminated patients from controls with an AUC above 0.7, but it was not among the markers that distinguished antidepressant responders from nonresponders [62]. In treatment-resistant depression, lower pretreatment serum FGF-2 was associated with response to a single ketamine infusion in an exploratory multiplex analysis of 33 patients [63]. In the larger CO-MED trial (*n* = 166), baseline plasma basic FGF did not predict differential response to bupropion–escitalopram, escitalopram alone, or venlafaxine–mirtazapine [64]. In outpatients with MDD, serum FGF did not differ from controls as a main effect, but the interaction between MDD and metabolic syndrome was associated with lower FGF, indicating that metabolic status may confound peripheral measurements [65]. Wang et al. (2023) found that baseline plasma basic FGF was higher in patients with MDD who had residual subjective cognitive decline after eight weeks of treatment than in those who did not [66].

However, other findings limit its clinical applicability. He et al. (2014) found no association between serum FGF-2 levels and disease severity or treatment response [33]. This may reflect inconsistency in peripheral FGF-2 measurements, whereas brain tissue studies more consistently report reduced FGF-2 levels in depression. Thus, circulating FGF-2 may not reliably reflect central nervous system alterations.

Pharmacogenomic evidence suggests additional clinical relevance. In 144 Japanese patients with MDD treated with paroxetine or fluvoxamine for six weeks, carriers of the rs1449683 T and rs308393 C alleles of the *FGF2* gene showed a better response, whereas rs1048201 was associated with dropout due to side effects [67]. In a subsequent analysis of two pooled trials from the same group (168 patients), the *FGF2* rs1449683 genotype, together with early partial improvement at two weeks, contributed to the prediction of week-6 outcome in the fluvoxamine arm (*n* = 42), but not in the paroxetine or milnacipran arms [68].

These analyses were exploratory, uncorrected for multiple comparisons, and unreplicated. Overall, FGF-2 may have value in biomarker panels and genetic profiling for personalized antidepressant treatment, but its independent clinical utility requires further validation.

### 3.2. Fibroblast Growth Factor 9 (FGF-9)

Fibroblast growth factor 9 (FGF-9) is a member of the FGF family that has increasingly been implicated in the pathophysiology of major depressive disorder (MDD). In contrast to FGF-2, which is generally associated with neuroprotective and antidepressant-like effects, FGF-9 appears to exert anxiogenic and prodepressant actions [44]. FGF-9 is expressed predominantly by neurons in the cortex, hippocampus, thalamus, cerebellum, and spinal cord, where it contributes to cell survival and inhibits astrocyte differentiation [10]. At the molecular level, FGF-9 signals preferentially through fibroblast growth factor receptor 3 (FGFR3), especially the adult IIIc splice variant, suggesting that altered FGF-9/FGFR3 signaling may be relevant to the neurobiological mechanisms underlying depression [69]. Recent investigations have positioned FGF-9 as the first growth factor described as having a sustained anxiogenic and prodepressant role in the hippocampus, acting in physiological opposition to the protective effects of FGF-2 [44].

Human postmortem studies have reported elevated FGF-9 expression in several brain regions implicated in mood regulation. In a study conducted by Aurbach et al. (2015), FGF-9 expression in the hippocampus was found to be 32% higher in individuals with MDD than in non-psychiatric controls, with a moderate effect size (Cohen’s d = 0.57) [44]. This finding was replicated across three independent microarray analyses and subsequently validated using quantitative RT-PCR [44]. Beyond the hippocampus, increased FGF-9 expression in individuals with MDD has been reported in the dorsolateral prefrontal cortex and anterior cingulate cortex [43]. Aurbach et al. (2015) also found that higher hippocampal FGF-9 expression in individuals with MDD was accompanied by lower expression of FGF-2 and its receptors FGFR1, FGFR2, and FGFR3 [44]. This opposing pattern further supports the hypothesis that FGF-9 may contribute to depressive pathology, whereas FGF-2 signaling may exert protective effects.

Evidence from peripheral biomarker research further supports the involvement of FGF-9 in depression. Wang et al. (2020) reported significantly elevated plasma FGF-9 levels in patients with depression compared with non-depressive controls [70]. In the same study, the adiponectin-to-FGF-9 ratio was negatively correlated with scores on the Hamilton Depression Scale, suggesting that this ratio may reflect depression severity more closely than FGF-9 levels alone. Additional clinical evidence comes from a case report by Faugère et al. (2025), which described treatment-resistant depression in a patient with Muenke syndrome caused by an *FGFR3* mutation, thereby indicating a possible association between altered FGF-9/FGFR3 signaling and depressive pathology [71].

Building on the associations observed in human studies, preclinical research has examined whether altered FGF-9 signaling may contribute directly to depression-related phenotypes. In a rodent model of chronic social defeat stress, Aurbach et al. (2015) found significantly increased FGF-9 expression in the hippocampus, consistent with the elevated hippocampal expression previously identified in postmortem brain tissue from individuals with MDD [44]. The same study further demonstrated that chronic intracerebroventricular administration of FGF-9 increased anxiety- and depression-like behaviors in rodents, including greater anxiety in the elevated plus maze and increased immobility in the forced swim test. Conversely, lentiviral knockdown of FGF-9 specifically within the dentate gyrus reduced anxiety-like behavior, providing experimental evidence that elevated FGF-9 activity may contribute to negative affective phenotypes [44]. Further evidence was provided by Wang et al. (2020) using the chronic unpredictable mild stress (CUMS) model [70]. In stressed mice, FGF-9 levels were elevated in both the plasma and hippocampus, while administration of an anti-FGF-9 antibody into the lateral ventricle attenuated depressive-like behaviors. This intervention also reversed the CUMS-induced reduction in hippocampal FGFR3 protein expression. In the same study, adiponectin was identified as a negative regulator of FGF-9/FGFR3 signaling: adiponectin knockout mice showed greater susceptibility to depressive-like behaviors, which was alleviated by anti-FGF-9 treatment. Together, these findings support the involvement of the adiponectin–FGF-9/FGFR3 regulatory axis in stress-induced depressive-like phenotypes [70].

The same group subsequently reported that carnosic acid, a rosemary-derived diterpene, attenuated depressive-like behavior in mice exposed to chronic unpredictable mild stress and attenuated the stress-induced increase in serum and hippocampal FGF-9, together with the decrease in adiponectin [72]. In adiponectin-deficient mice, carnosic acid no longer improved depressive-like behavior or reduced serum and hippocampal FGF-9, supporting an adiponectin-dependent mechanism [72]. Elevated FGF-9 has also been observed in a rat model of post-stroke depression. FGF-9 protein and mRNA in the dentate gyrus were increased after middle cerebral artery occlusion alone and further increased when occlusion was combined with chronic mild stress. FGFR3 was reduced in both groups, whereas FGFR1 was reduced only in rats with post-stroke depression. Fluoxetine partly reversed these alterations [73].

In mice exposed to chronic unpredictable mild stress, both fluoxetine and swimming exercise reversed depressive-like behavior and the stress-induced dysregulation of hippocampal *Fgf9*, *Fgf2*, and *FgfR2* expression, and these effects were associated with changes in Fgf9/Akt signaling [74].

In summary, the evidence for a prodepressant role of FGF-9 is derived almost entirely from rodent studies, in which FGF-9 administration, knockdown, or neutralization altered depression-like behavior. Human data are limited to increased FGF-9 transcript expression in postmortem tissue, one plasma study, and one case report, none of which establishes the direction of the relationship. The contrast between FGF-2 and FGF-9 should therefore be regarded as a hypothesis generated in animal models rather than a finding established in patients.

### 3.3. Other Paracrine FGF Ligands with Emerging Evidence (FGF-17 and FGF-20)

FGF17 is a secreted ligand of the FGF8 subfamily with established roles in brain development, including patterning of the frontal cortex [15,75]. Its relationship to depression has only recently been examined. In a cross-sectional study, Zhu et al. compared serum FGF17 levels between antidepressant-free patients with MDD and healthy controls and found lower levels in patients. Within the MDD group, lower FGF17 was associated with more severe negative-like symptoms, including deficits in the motivation–pleasure and expression domains assessed with the Clinical Assessment Interview for Negative Symptoms (CAINS). After adjustment for demographic and clinical covariates and correction for multiple comparisons, FGF17 remained significantly associated with the expression domain and total CAINS scores, whereas the association with the motivation–pleasure domain did not remain significant. These preliminary findings suggest an association between reduced peripheral FGF17 and specific negative-like symptom dimensions of depression, although the cross-sectional design precludes conclusions about causality [76].

Genetic studies have also suggested a potential role of FGF20 in depression-related phenotypes. FGF20 is a secreted ligand of the FGF9 subfamily with neurotrophic actions [15], and a functional polymorphism in the 3′ untranslated region of its gene (rs12720208) reduces microRNA-433-mediated repression, thereby increasing FGF20 translation [77]. In a community sample of young adults, Jiménez et al. reported that carriers of the minor A allele of this variant had higher depressive-symptom scores than G/G homozygotes. This was the first reported association between a functional *FGF20* polymorphism and depressive symptoms, although replication is needed, particularly in patients with MDD [78].

### 3.4. Endocrine Fibroblast Growth Factors (FGF-19, FGF-21, and FGF-23)

FGF19, FGF21, and FGF23 belong to the endocrine FGF subfamily, which signals through FGFRs in complex with Klotho co-receptors and acts systemically as circulating hormones (Figure 3) [11,16]. FGF19 and FGF21 have been examined in depression mainly in the context of the metabolic comorbidity of MDD, whereas FGF23 has emerged only recently from multiplex inflammatory protein panels.

Fibroblast growth factor 21 (FGF-21) is an endocrine regulator of metabolic homeostasis and nutrient preference that may also modulate biological pathways implicated in major depressive disorder (MDD), including corticotropin-releasing hormone (CRH) signaling, leptin-related mechanisms, and sympathetic nervous system activity [79].

Evidence regarding FGF-21 in depression remains limited. Mason et al. (2022) measured FGF21 using ELISA in venous blood samples from participants with chronic, early-onset major depressive disorder (MDD) enrolled in Stage 1 of the EMBARC trial [79]. The final analytical sample comprised 207 participants with MDD and 32 healthy controls. Baseline FGF21 concentrations were significantly higher in the MDD group than in healthy controls. This difference remained significant after adjustment for age, sex, race, Hispanic ethnicity, BMI, and study site (β = 1.20; *p* < 0.0001, Cohen’s d = 0.60). FGF21 concentrations did not change significantly over the analyzed 8-week treatment phase and did not differ between the sertraline and placebo groups. A significant interaction between baseline FGF21, BMI, and changes in depressive symptom severity was reported: among participants with normal BMI, lower baseline FGF21 concentrations were associated with a greater reduction in HAMD-17 scores over time, whereas no such difference was observed in participants with overweight or obesity [79].

In contrast, Liu et al. (2017) measured cerebrospinal fluid (CSF) fibroblast growth factor 21 (FGF21) concentrations in 67 Chinese volunteers, in whom depressive symptoms were assessed using the self-reported Beck Depression Inventory (BDI) [80]. The study did not report a formal diagnosis of major depressive disorder (MDD), and all participants had BDI scores below 17. Lower CSF FGF21 concentrations were significantly associated with higher BDI scores in male participants (r = −0.440, *p* = 0.012), but not in females (r = 0.081, *p* = 0.664) [80].

Conversely, Tang et al. (2023) conducted a case–control study including 59 first-diagnosed, drug-naive patients with major depressive disorder (MDD) and 55 healthy controls [81]. Circulating FGF21 levels were significantly lower in patients with MDD than in healthy controls (99.84 ± 53.69 vs. 165.17 ± 111.81; *p* < 0.01). In healthy controls, FGF21 levels were positively correlated with Beck Depression Inventory-II (BDI-II) scores (r = 0.5184, *p* = 0.0014), whereas no significant associations between FGF21 and clinical symptom scores were observed in patients with MDD.

Wu et al. (2021) examined serum FGF21 levels in 116 patients with stable coronary artery disease (SCAD), including 86 with and 30 without depressive symptoms, and 45 healthy controls [82]. Depressive symptoms were assessed using the Zung Self-Rating Depression Scale (SDS), rather than a formal psychiatric diagnosis. FGF21 levels were lower in patients with SCAD than in healthy controls; however, within the SCAD group, patients with depressive symptoms had higher FGF21 levels than those without depressive symptoms (both *p* = 0.039). FGF21 was not significantly associated with SDS scores in regression analysis (*p* = 0.217).

Two recent studies in populations not selected for clinically diagnosed MDD point toward a positive association between FGF21 and depressive symptom burden. Among 1160 adults with type 1 or type 2 diabetes, Herder et al. found a positive association between serum FGF21 and depressive symptoms measured with the Center for Epidemiologic Studies Depression Scale. The association persisted after adjustment for demographic, metabolic, and pharmacological covariates and did not differ by diabetes type. The analysis was exploratory and was not corrected for multiple testing [83]. In a subgroup of 52 community-dwelling older adults from the FRASNET cohort, Damanti et al. reported higher plasma FGF21 concentrations in participants screening positive for depressive symptoms, and higher baseline FGF21 was associated with greater depressive symptom severity at follow-up after adjustment for age, sex, and BMI. Because depressive symptoms were assessed using the Geriatric Depression Scale rather than a clinical diagnosis and only a small number of participants screened positive, the authors considered these findings preliminary and hypothesis-generating [84].

Two rodent studies support a protective role of FGF21 signaling in inflammation- and stress-induced depressive-like behavior. Wang et al. showed that lipopolysaccharide reduced endogenous FGF21 expression in the mouse hippocampus and that pretreatment with recombinant FGF21 attenuated depressive-like behavior, microglial activation, and proinflammatory cytokine expression, while suppressing NF-κB signaling and restoring hippocampal FGFR1 phosphorylation and BDNF expression. In primary microglia, inhibition of FGFR1 reversed the anti-inflammatory and BDNF-related effects of FGF21, supporting an FGFR1-dependent mechanism; recombinant FGF21 had no significant behavioral effect in unchallenged mice [85]. Usui et al. found that social defeat stress increased plasma FGF21 concentrations and that a subset of catecholaminergic neurons in the nucleus tractus solitarius and area postrema expressed β-Klotho. FGF21-deficient mice displayed greater social avoidance and longer immobility in the forced swim test after social defeat, whereas viral overexpression of FGF21 did not alter these behaviors in either wild-type or FGF21-deficient mice. The authors therefore proposed that endogenous FGF21 may restrain stress-induced depressive-like behavior, possibly through effects established during development [86].

Taken together, preclinical studies support a protective role of FGF21 signaling: exogenous FGF21 attenuates inflammation-induced depressive-like behavior, whereas endogenous FGF21 appears to restrain behavioral responses to social stress. Clinical findings, however, are inconsistent in direction. Case–control studies of MDD have reported both higher and lower circulating FGF21, whereas a large cross-sectional study in diabetes and a small prospective study in older adults both associated higher FGF21 with greater depressive symptom burden. Because circulating FGF21 is strongly influenced by obesity, insulin resistance, and metabolic stress, elevated concentrations in individuals with depressive symptoms may reflect metabolic comorbidity or a compensatory adaptive response rather than a direct pathogenic contribution. This interpretation is consistent with the modifying effect of BMI reported by Mason et al. and with the counterregulatory role proposed in recent clinical studies. The nature of this relationship remains to be established in longitudinal studies that account for metabolic status.

FGF19 is an endocrine FGF secreted by the ileum in response to bile-acid activation of the farnesoid X receptor and signals through FGFRs in complex with the co-receptor β-Klotho [87,88]. FGF19 has also been detected in human cerebrospinal fluid [89].

Four cross-sectional analyses have evaluated FGF19 in relation to MDD or depressive symptoms. Liu et al. reported a positive correlation between cerebrospinal fluid FGF19 concentrations and Beck Depression Inventory scores in 83 Chinese men recruited as controls for a study of nicotine dependence [89]. Tang et al. reported significantly lower serum FGF19 concentrations in drug-naive patients with a first diagnosis of MDD than in healthy controls; within the patient group, however, FGF19 concentrations were positively correlated with symptom severity on both clinician-rated and self-report scales. These associations were derived from unadjusted bivariate analyses and therefore warrant cautious interpretation [81]. Li et al. subsequently reported a similar association in 156 Chinese men, including smokers; the association between CSF FGF19 and Beck Depression Inventory scores was present in non-smokers but absent in smokers, and moderation analysis demonstrated a significant modifying effect of smoking status [90]. In the largest analysis, comprising 831 participants of the Framingham Offspring cohort, plasma FGF19 was positively associated with depressive-symptom scores after correction for multiple testing and adjustment for demographic, metabolic, and pharmacological covariates. The association was strongest for the somatic-complaints subscale. It reached significance in men but not in women, although the authors found no significant difference between sexes. No association was observed when depressive symptoms were analyzed categorically; the relatively small number of participants above the cutoff limited statistical power [91].

In summary, all four analyses found that higher FGF19 concentrations were associated with greater depressive-symptom severity within the studied samples, both in blood and in cerebrospinal fluid. In contrast, the only comparison between patients with MDD and healthy controls found lower serum FGF19 in patients. The basis of this discrepancy remains unclear and may reflect differences between diagnostic-group comparisons and within-sample symptom associations, compensatory regulation, or modification by smoking status. FGF19 therefore remains a candidate marker requiring confirmation in larger clinical and longitudinal studies.

FGF23 is an endocrine FGF produced mainly by osteocytes that regulates renal phosphate handling and vitamin D metabolism, signaling through FGFR1 in complex with the co-receptor α-Klotho [92,93]. Three studies have examined FGF-23 in relation to depression, none of them in patients with clinically diagnosed MDD compared with healthy controls.

Two observational studies associated higher plasma FGF-23 with depressive symptoms. Among 169 Swedish women assessed eight weeks postpartum, FGF-23 was one of five proteins, out of 70 analyzed, associated with postpartum depressive symptoms in an adjusted penalized regression model (odds ratio 1.25 per one standard deviation), although no marker survived Bonferroni correction in conventional regression [94]. In 61 patients with inflammatory bowel disease, FGF-23 was higher in those with anxiety or depressive symptoms and, separately, in the subgroup with depressive symptoms [95]. It correlated with total and anxiety subscale scores of the Hospital Anxiety and Depression Scale but not with the depression subscale, and no correction for multiple comparisons was applied. FGF-23 was not associated with intestinal disease activity, but systemic inflammation, renal function, and mineral metabolism were not accounted for.

In contrast, a two-sample Mendelian randomization study of 95 circulating inflammatory proteins found that genetically predicted higher FGF23 was associated with a slightly lower risk of MDD in the discovery sample (odds ratio 0.96 per standard deviation), in a meta-analysis with FinnGen (odds ratio 0.97), and after adjustment for other inflammatory proteins [96]. The estimate was significant only with the inverse-variance weighted method, did not survive correction for false discovery rate, and was not supported by colocalization.

The observational and genetic findings therefore point in opposite directions. Elevated FGF-23 in symptomatic individuals may reflect inflammation, renal or mineral-metabolic changes, or reverse causation, whereas the weak protective genetic signal, if confirmed, would argue against a harmful effect of lifelong higher FGF23 exposure. Studies in clinically diagnosed MDD, with adjustment for renal function and inflammatory status, are required before any conclusion can be drawn.

### 3.5. Fibroblast Growth Factor 22 (FGF-22)

Current evidence linking FGF-22 to depression remains very limited but suggests a potential role in developmental pathways involved in affective regulation. Preclinical studies indicate that FGF-22 deficiency is associated with depression-like phenotypes in mice. In constitutive knockout models, loss of FGF-22 was associated with despair-like behavior in the forced swim and tail suspension tests, as well as anhedonia-like behavior in the sucrose preference test. These behavioral alterations may be partly attributable to impaired FGF-22–FGFR2 signaling and reduced formation of excitatory synapses in the hippocampus [97]. Subsequent conditional knockout studies further refined this developmental interpretation, showing that neonatal, but not adult, neuronal FGF-22 inactivation induces anhedonia, indicating that FGF-22 signaling during development is particularly relevant to hedonic regulation [98]. The authors further suggested that defects in growth factor-dependent synapse development and/or neurogenesis may contribute to depression of developmental origin. Clinical evidence, although preliminary, supports an association between FGF-22 and depressive symptom severity: serum FGF-22 levels were significantly reduced in 90 first-onset depressive patients compared with healthy volunteers, negatively correlated with Hamilton Depression Scale scores, and increased after 8 weeks of antidepressant treatment [99]. FGF-22 has also been broadly associated with depression across species and functions as a paracrine factor expressed primarily in brain and skin tissues [100]. However, the available evidence remains insufficient for definitive conclusions, as only one clinical dataset is currently available and no studies have examined exogenous FGF-22 administration for therapeutic purposes. Further replication and translational studies are therefore required to establish the clinical relevance of FGF-22 in depression.

### 3.6. Fibroblast Growth Factors 13 and 14 (FGF13 and 14)

Unlike canonical and endocrine FGFs, fibroblast homologous factors (FHFs; FGF11–FGF14) lack a secretory signal peptide and function intracellularly, independently of FGFR activation. Their association with depression, therefore, suggests the involvement of mechanisms distinct from classical paracrine FGF–FGFR signaling [15].

*FGF13* has recently emerged as a candidate gene associated with depression. In a study by Hu et al., weighted gene co-expression network analysis of peripheral-blood transcriptomic data identified gene modules linked to depression, with subsequent network analysis highlighting *FGF13* as a highly connected hub gene [101].

The authors subsequently confirmed reduced FGF13 expression in experimental models of depression-related pathology: FGF13 was decreased in corticosterone-treated PC12 (rat pheochromocytoma) and SH-SY5Y (human neuroblastoma) cells, both used as neuronal models, and in the hippocampus of rats exposed to chronic unpredictable mild stress (CUMS). Overexpression of FGF13 increased the levels of synaptic proteins (synapsin and PSD95) and BDNF, together with the anti-apoptotic protein Bcl-2, decreased the pro-apoptotic proteins Bax and cleaved caspase-3, and enhanced PI3K/AKT signaling; the PI3K inhibitor LY294002 markedly attenuated these effects, indicating that they are mediated, at least in part, through the PI3K/AKT pathway [101].

In vivo, hippocampal FGF13 overexpression reversed CUMS-induced changes in sucrose preference, locomotor activity, and forced-swim immobility, while also reducing hippocampal apoptosis. In a clinical cohort of 30 patients with MDD and 30 healthy controls, serum FGF13 levels were significantly lower in patients and showed high discriminatory performance (AUC = 0.936; 95% CI 0.880–0.992). Higher FGF13 levels were also associated with a lower probability of MDD [101].

A subsequent study by Zheng et al. linked FGF13 to astrocyte survival in depression. Reanalysis of a publicly available single-nucleus RNA-sequencing dataset from postmortem dorsolateral prefrontal cortex showed that FGF13 expression was selectively reduced in astrocytes from individuals with MDD. Consistently, hippocampal FGF13 expression was decreased in mouse models of chronic restraint stress and chronic social defeat stress, accompanied by astrocyte loss and apoptosis. Astrocyte-specific deletion of *FGF13* promoted astrocytic apoptosis, increased inflammatory responses, and induced depression-like behaviors, whereas astrocyte-specific overexpression produced the opposite effects. Mechanistically, FGF13 was shown to bind JIP2 and inhibit downstream JIP2–JNK signaling, thereby limiting Bax/Bcl-2-mediated astrocyte apoptosis [102].

FGF14 is another member of the intracellular FHF subfamily, but it acts through a distinct mechanism. As an accessory protein of voltage-gated sodium (Nav) channels, it regulates their localization at the axon initial segment and controls neuronal excitability [15]. The FGF14–Nav complex is regulated by GSK3 [103], a kinase implicated in the pathophysiology of mood disorders [104], while *FGF14* has also emerged as a candidate gene in genetic studies of MDD [105].

Its functional role was examined in female *Fgf14*-knockout (*Fgf14*−/−) mice. In contrast to the generally protective effects described for secreted FGFs, loss of FGF14 conferred resilience: knockout mice showed reduced despair-like and anxiety-like behavior, less anhedonia, and increased sociability. Importantly, locomotor activity did not differ between genotypes, making it unlikely that these behavioral effects were secondary to the motor deficits of *Fgf14*−/− mice. This resilience was associated with selective changes in the medial prefrontal cortex (mPFC) rather than with a generalized increase in neuronal activation: *Fgf14*−/− mice showed a dissociation in cannabinoid receptor 1 expression (increased mRNA but reduced protein), and acute stress selectively recruited non-GABAergic, presumably pyramidal, neurons. Because the FGF14–Nav complex is regulated by GSK3 and GSK3 inhibition produces antidepressant-like effects in animal models, these findings raise the possibility that reduced FGF14 function may promote resilience to depression-like behavior through a mechanism distinct from the neurotrophic and anti-apoptotic actions described for secreted FGFs and FGF13 [106].

### 3.7. Fibroblast Growth Factor Receptor 1 (FGFR1)

Fibroblast growth factor receptor 1 (FGFR1) represents an important receptor-level component of FGF signaling in depression-relevant neural circuits. Its relevance is supported by studies implicating FGFR1 in hippocampal development, long-term synaptic plasticity, and broader neurodevelopmental processes [28,107,108]. Because hippocampal and prefrontal networks are central to affective regulation, alterations in FGFR1 signaling have been investigated within the broader neurotrophic framework of major depressive disorder.

The postmortem literature on FGFR1 expression in depression is limited and inconsistent and appears to depend on the brain region examined. In the early microarray analysis of the FGF system in major depression, *FGFR1* transcript was reliably detected but not significantly altered in the dorsolateral prefrontal cortex or anterior cingulate cortex; among the FGF receptors, only *FGFR2* and *FGFR3* were dysregulated [43]. In contrast, Gaughran et al. reported increased *FGFR1* mRNA expression in the hippocampal CA1, CA4, and dentate regions in patients with non-psychotic major depression, together with reduced *FGF2* mRNA expression in CA4 [42], and increased *FGFR1* gene expression was subsequently described in the prefrontal cortex [109,110]. Notably, among studies detecting significant alterations, human postmortem evidence generally points to increased rather than decreased FGFR1 expression, particularly within hippocampal regions.

Rodent studies suggest that FGFR1 expression is modulated by both stress exposure and ligand-specific FGF signaling. Acute social defeat in rats was associated with reduced hippocampal *FGFR1* mRNA expression [111], whereas intracerebroventricular administration of FGF2 increased *FGFR1* mRNA in the dentate gyrus and produced antidepressant-like behavioral effects [56]. In contrast, exogenous FGF9, which has been linked to anxiety- and depression-like behavior, reduced FGFR1 expression in the dentate gyrus [44]. In interleukin-18-deficient mice, which display depressive-like behavior, whole-brain microarray profiling identified *Fgfr1* among 13 depression-associated genes, with increased expression at 12 weeks of age that the authors interpreted as compensatory to reduced neurogenesis [112]. The increase was not confirmed by RT-qPCR for this gene, and expression was not resolved by region.

Together, these findings suggest that FGFR1 signaling is state-sensitive and may be influenced by the balance between FGF ligands with opposing behavioral profiles.

A key mechanistic theme concerns the interaction between FGFR1 and serotonergic signaling. FGFR1 can form heteroreceptor complexes with 5-HT1A receptors within the raphe–hippocampal serotonin system [113,114,115]. Through this interaction, FGFR1 activation has been proposed to modulate 5-HT1A-dependent GIRK channel signaling and thereby reduce serotonergic neuronal inhibition. In Flinders Sensitive Line rats, a genetic model of depression, this receptor–receptor modulation appears impaired, and combined FGF2 plus 5-HT1A agonist treatment fails to produce the antidepressant-like response observed in control animals [116,117]. This mechanism offers a point of convergence between neurotrophic and monoaminergic models of depression.

FGFR1-related signaling is also implicated in neuroplastic and gliogenic mechanisms relevant to antidepressant response, although some of this evidence concerns FGF/FGFR signaling more broadly rather than FGFR1 alone. FGF2–FGFR signaling has been linked to hippocampal neurogenesis, neuronal survival, synaptic remodeling, and glial proliferation, processes frequently implicated in depression pathophysiology [58,69]. At the transcriptomic level, a cross-species analysis of hippocampal expression profiles in two rat models of depression and in postmortem samples from patients with MDD identified FGFR-related signaling, including pathways involving FGFR1, among 67 pathways significantly enriched in both rat and human signature networks [118].

In preclinical models, pharmacological inhibition of FGFR signaling blocked antidepressant-like behavioral effects and prevented antidepressant-induced gliogenic responses, supporting the view that FGFR signaling is required for at least some of the cellular and behavioral effects of antidepressant treatment [58]. FGFR signaling has also been probed independently of canonical FGF ligands. In NCAM-deficient mice, which exhibit despair-like and anhedonia-like behavior together with reduced dentate neurogenesis, the NCAM-derived FGFR-activating peptide FGL reduced depression-like behavior after both acute and repeated administration and enhanced the survival of newborn neurons, whereas these effects were absent in wild-type littermates [119].

Consistent with this framework, *Fgf2*-knockout mice failed to show the expected behavioral response to fluoxetine, suggesting that intact FGF2-dependent signaling is necessary for fluoxetine efficacy in this model [55].

Shin et al. recently linked FGFR1 signaling in the dentate gyrus with major depressive disorder. The authors reported increased FGFR1 expression in postmortem dentate gyrus samples from patients with MDD and showed, using in vivo optogenetic approaches, that FGFR1 activation engages a Notch–BDNF pathway involved in adult hippocampal neurogenesis and antidepressant-like effects. They also identified Numb as an age-related inhibitor of this pathway, with increased Numb expression associated with disrupted FGFR1–Notch–BDNF signaling and depressive-like phenotypes; targeting Numb restored pathway activity and reversed the depressive phenotype [120].

Additional preclinical evidence links FGFR1 activation to the anti-inflammatory effects of FGF21 in depression-related models. In an LPS-induced mouse model, recombinant human FGF21 improved depressive-like behaviors, reduced hippocampal microglial activation, suppressed pro-inflammatory cytokines, and inhibited NF-κB signaling. These protective effects were significantly reversed by coadministration of the FGFR1 inhibitor PD173074, suggesting that the antidepressant-like and anti-inflammatory effects of FGF21 in this model depend, at least in part, on FGFR1 activation [85].

Overall, FGFR1 appears to be a relevant component of FGF signaling in depression, with evidence linking it to hippocampal plasticity, serotonergic regulation, and antidepressant-related neurogenic mechanisms. However, the direction of FGFR1 changes differs across models and tissue contexts, suggesting dysregulated rather than uniformly decreased or increased signaling.

### 3.8. Fibroblast Growth Factor Receptors 2, 3 and 4 (FGFR2, FGFR3, and FGFR4)

FGFR2 and FGFR3 have been examined only to a limited extent in depression, with most evidence derived from postmortem studies. In postmortem prefrontal cortex, Goswami et al. found no change in FGFR2, FGFR3, or FGFR4 expression in patients with major depressive disorder [110]. In contrast, two human postmortem studies reported reduced FGFR2 and FGFR3 expression in depression: Evans et al. found decreased expression in the dorsolateral prefrontal cortex and anterior cingulate cortex of depressed subjects [43], whereas Aurbach et al. reported decreased expression in the hippocampus of postmortem MDD subjects [44]. In the locus coeruleus, altered expression of *FGFR3* was reported among growth-factor genes dysregulated in MDD but not in bipolar disorder [121].

Additional experimental evidence implicates FGFR2 in antidepressant-related plasticity, as Carboni et al. reported that nortriptyline increased *Fgfr2* expression in Flinders Sensitive Line rats and hippocampal neurons and described a Negr1–Fgfr2 pathway involved in dendritic spine remodeling [122]. Overall, current evidence suggests possible region-dependent alterations of FGFR2 and FGFR3 in depression, whereas evidence for FGFR4 remains limited to negative postmortem findings in the prefrontal cortex [110].

## 4. Conclusions and Future Directions

Several factors may contribute to the heterogeneity of the available findings. These include differences in diagnostic criteria, sample size, sex distribution, pharmacological treatment status, disease duration, severity of depressive symptoms, and the biological material analyzed. Moreover, depression rarely occurs as a biologically uniform condition. Psychiatric comorbidities, such as anxiety disorders, personality disorders, substance use disorders, or trauma-related conditions, may significantly influence neurotrophic signaling and should be carefully considered in future studies. It is also plausible that FGF-related alterations differ across clinical subtypes of depression, including late-life depression, treatment-resistant depression, recurrent depression, and depression associated with chronic stress or somatic illness. Future research should aim to include more precisely characterized clinical groups, control for psychiatric and somatic comorbidities, and distinguish between central and peripheral FGF-related changes.

### Limitations

This review has several limitations. Only two databases were searched, and backward citation searching identified eleven additional eligible studies, suggesting that the database search may not have captured all the relevant literature. No review protocol was registered, although the search strategy and eligibility criteria were specified before screening began.

Four studies could not be assessed in full text and were therefore evaluated on the basis of abstracts only (Xia 2021, Jha 2017, Xu 2023, Maes 2024), as indicated in Table S1, limiting the completeness and certainty of data extraction [62,64,65,74]. The included studies were highly heterogeneous in design, population, tissue or biological compartment, and measurement methods, which precluded meaningful quantitative synthesis. Two cerebrospinal fluid studies of FGF19 appear to have been based on overlapping samples, potentially limiting the independence of these findings. Finally, interpretation of peripheral FGF concentrations is constrained by the uncertain relationship between circulating and central FGF levels and by potential confounding from metabolic and inflammatory factors.

## Figures and Tables

**Figure 1:**
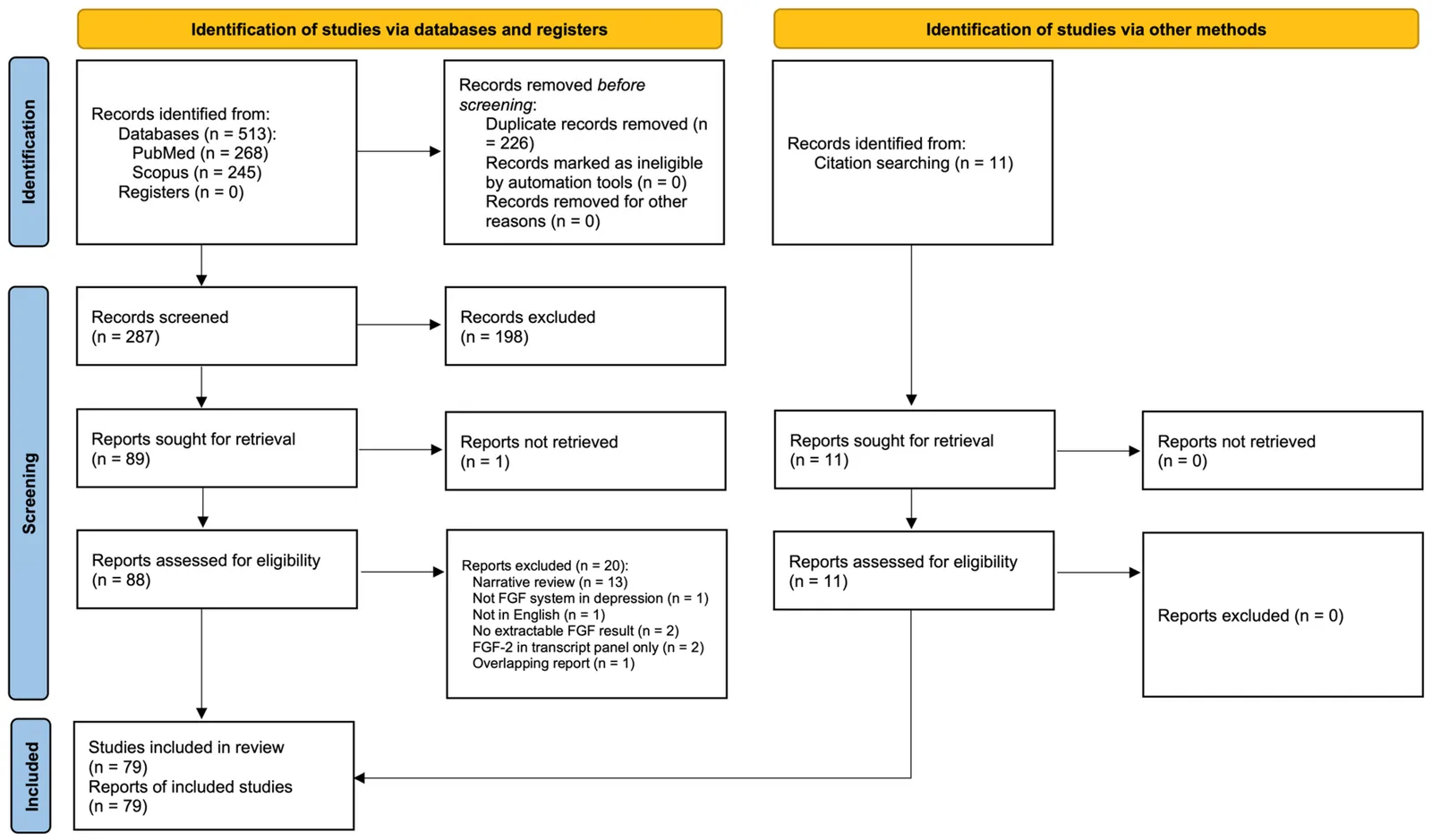
PRISMA 2020 flow diagram of the study selection process.

**Figure 3:**
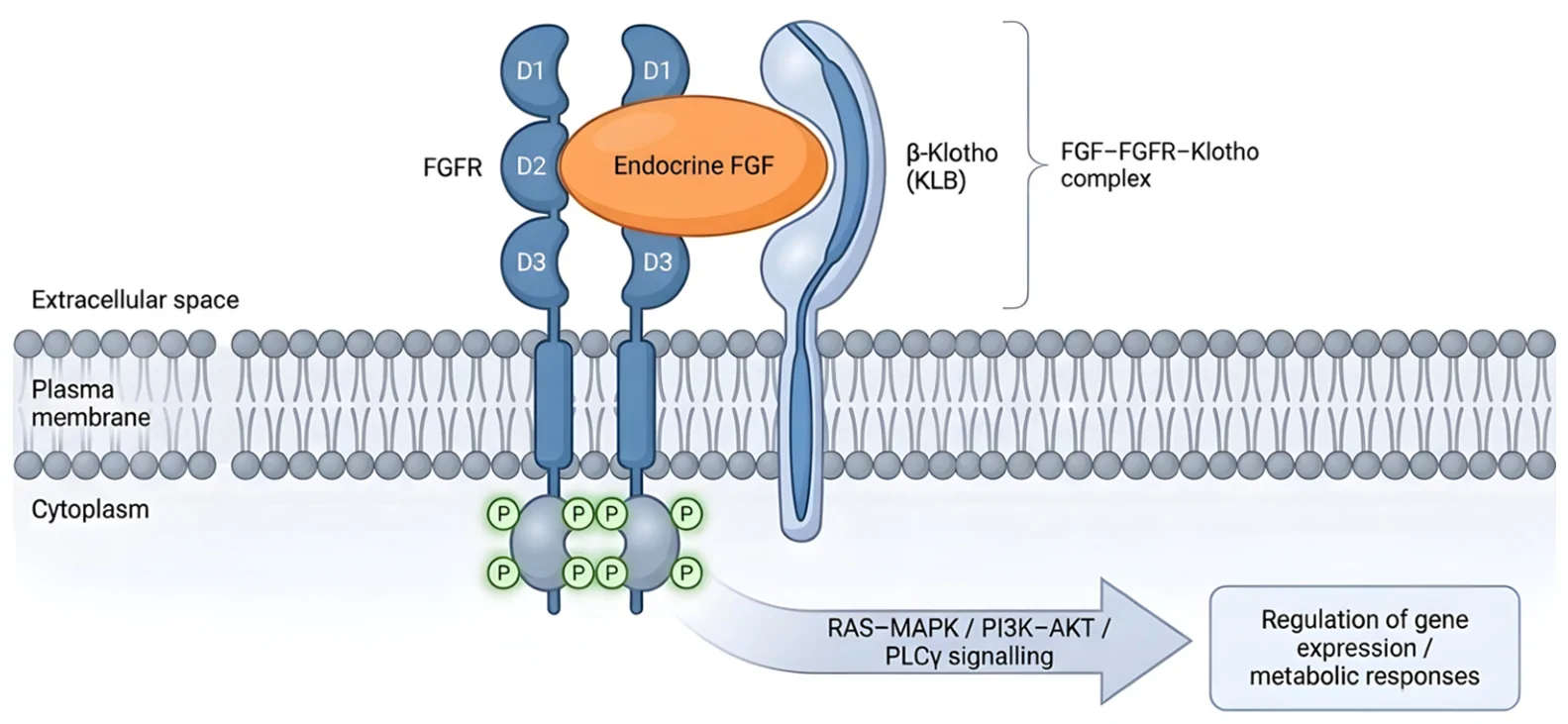
**Endocrine FGF signaling via the β-Klotho co-receptor.** In contrast to canonical (paracrine) FGFs, endocrine FGFs (FGF19, FGF21 and FGF23) have low affinity for heparan sulfate and instead require a Klotho-family co-receptor to bind FGFRs with high affinity. β-Klotho (KLB) acts as the co-receptor for FGF19 and FGF21 (α-Klotho for FGF23), forming an FGF–FGFR–Klotho complex that drives receptor dimerization and trans-autophosphorylation of the intracellular tyrosine kinase domains (P). This activates the same downstream cascades as canonical FGF signaling (RAS–MAPK, PI3K–AKT and PLCγ), regulating gene expression and systemic metabolic responses. The requirement for Klotho underlies the endocrine, hormone-like mode of action of these FGFs. Prepared by the authors on the basis of the mechanisms described in Ornitz and Itoh (2015) [15].

## Data Availability

No new data were created or analyzed in this study. Data sharing is not applicable to this article.

## Supplementary Materials

The following supporting information can be downloaded at: https://www.mdpi.com/article/10.3390/ijms27198464/s1, References [30,31,32,33,34,35,36,37,38,39,40,41,42,43,44,45,46,47,48,49,50,51,52,53,54,55,56,57,58,59,60,61,62,63,64,65,66,67,68,70,71,72,73,74,76,78,79,80,81,82,83,84,85,86,89,90,91,94,95,96,97,98,99,101,102,106,109,110,111,112,114,115,116,117,118,119,120,121,122] are cited in the supplementary materials.
